# Safety and Efficacy of the Use of Supraglottic Airway Devices in Children and Adolescents Undergoing Adenotonsillectomy—A Systematic Review and Meta-Analysis

**DOI:** 10.3390/jpm14030311

**Published:** 2024-03-16

**Authors:** Abhijit Nair, Nitinkumar Borkar, Sunil Subhash Murke, Ujjwalraj Dudhedia

**Affiliations:** 1 Department of Anesthesiology, Ibra Hospital, Ibra 413, Oman; 2 Department of Pediatric Surgery, All India Institute of Medical Sciences, Raipur 492001, India; drnitinborkar25@aiimsraipur.edu.in; 3 Department of Anesthesiology, Shri Ramchandra Institute of Medical Sciences, Aurangabad 431001, India; drmurkesunil@yahoo.co.in; 4 Department of Anesthesiology, Dr. L.H. Hiranandani Hospital, Mumbai 400001, India; ujjwalraj.dudhedia@hiranandanihospital.org

**Keywords:** anesthesia, adenoidectomy, laryngeal mask airway, supraglottic airway device, tonsillectomy

## Abstract

(1) Background: Supraglottic airway devices (SAD) have been used in children and adolescents undergoing adenotonsillectomies under general anesthesia. This systematic review and meta-analysis investigate the safety and efficacy of using SADs when compared to an endotracheal tube (ETT). (2) Methods: After registering with PROSPERO, databases like PubMed, Scopus, OviD, CINAHL, and Cochrane Library were searched using relevant keywords from the year 2000. We used RoB-2 for risk-of-bias assessment, GRADE for assessing the quality of evidence, RevMan 5.2 for qualitative meta-analysis, and trial sequential analysis (TSA) to corroborate the significant findings of meta-analysis. (3) Results: Out of 200 studies, 5 randomized-controlled trials fulfilled inclusion criteria. The quality of evidence was moderate for laryngospasm, low for airway device failure, and very low for recovery time. The incidence of laryngospasm was comparable between SADs and ETT (RR: 0.80, 95% CI-0.36, 1.80, *p* = 0.59). The incidence of airway device failure was significantly higher with SADs than ETT (RR: 11.29, 95% CI: 2.73, 46.66, *p* = 0.0008). The postoperative recovery time was significantly less with SADs than with ETT use (MD: −4.33, 95% CI: −5.28, −3.39, *p* < 0.0001), which was confirmed by the TSA. (4) Conclusions: The results of this review suggests that use of SADs can provide a lesser postoperative recovery time and comparable incidence of laryngospasm, with a higher incidence of failure of SAD when compared to ETT. Use of SAD for pediatric and adolescent adenotonsillectomies should be individualized based on patient characteristics, and on the expertise of the anesthesiologist and the surgeons involved.

## 1. Introduction

Adenoidectomy and tonsillectomy, either alone or as combined surgeries, are commonly performed surgical interventions in the pediatric population that necessitate meticulous airway management to ensure uninterrupted ventilation and to secure the airway during the surgery. Endotracheal intubation with an appropriate-sized tube has been the cornerstone for airway management during these surgeries [1,2,3]. Supraglottic airway devices (SADs), such as the laryngeal mask airway (LMA) and its various pediatric variations, when used as an airway adjunct, provide effective ventilation by forming a seal above the glottis, thereby obviating the need for tracheal intubation. The use of SAD reduces the incidence of trauma to the airway structures, subsequently avoiding the risk of postoperative complications [4,5]. A quantitative meta-analysis concluded that the use of SADs in pediatric anesthesia decreases the number of respiratory complications [6].

Recently, SAD has been used effectively with considerable safety as an alternative to endotracheal intubation in pediatric and adolescent patients undergoing adenotonsillectomy. Various studies have attested that SADs are less irritating to the otherwise reactive airways of these patients, thus reducing the incidence of perioperative respiratory adverse events like bronchospasm, laryngospasm, and incessant coughing postoperatively [7,8]. While the use of SADs in pediatric adenotonsillectomy is gaining popularity, anesthesiologists must be meticulous with patient selection, the type of SAD used, procedural technique, and ongoing monitoring. Appropriate sizing, correct placement, and vigilant perioperative monitoring are paramount to ensuring the efficacy and safety of SADs for this surgical indication [9,10].

The objective of this review was to investigate the safety and feasibility of using various SADs in pediatric and adolescent patients undergoing adenoidectomy, tonsillectomy, or both. We also aimed to evaluate other outcomes like success rate, various periods like induction time, recovery time, and time spent in the recovery room. 

## 2. Materials and Methods

This review adhered to the protocol established by the Preferred Reporting Items for Systematic Reviews and Meta-Analyses (PRISMA) guidelines, which comprise a checklist of 27 essential points. This systematic review was prospectively registered with PROSPERO (https://www.crd.york.ac.uk/PROSPERO/ (accessed on 27 December 2023)) with the registration number CRD42023494137.

### 2.1. Search Strategy

Starting from January 2000 until December 2023, we searched PubMed/Medline, Cochrane Library (CENTRAL), CINAHL, OViD, and Scopus using relevant keywords for randomized-controlled trials with the following keywords: supraglottic airway, laryngeal mask airway, adenoidectomy, tonsillectomy, adenotonsillectomy, pediatric, and adolescent. A detailed search strategy is presented in Table 1. Articles without a control group or not having the outcomes of interest were excluded from the analysis.

### 2.2. Eligibility Criteria

We used a structured Population, Intervention, Control, Outcome, Study (PICOS) design to select relevant studies. Only RCTs were included and studies not in English, animal studies, studies with unavailable full texts, systematic/literature/scoping reviews, case reports/series, editorials, and conference abstracts were excluded.

Population: all pediatric and adolescent patients undergoing adenoidectomy, tonsillectomy, or adenotonsillectomy were included.

Intervention: use of a SAD as an airway adjunct for general anesthesia.

Control: use of an endotracheal tube for general anesthesia.

Outcome: safety and efficacy of SAD for adenotonsillectomy.

Study: randomized-controlled studies.

### 2.3. Data Extraction

The initially identified studies were screened by two authors (AN and NB) according to the inclusion and exclusion criteria. If the two authors disagreed about the inclusion of the study, another author (UD) was asked to settle the disagreement. The primary outcome was the incidence of laryngospasm. Secondary outcomes were the incidence of airway device failure, recovery time, time spent in the operating room after surgery, and other adverse events like aspiration, regurgitation, and bronchospasm.

### 2.4. Data Collection

Two authors conducted data extraction for each included article and tabulated details like authors, year, participants, sample size, type of SAD used, outcomes, interventions, measurement instruments, key findings, and conclusions. The selection of studies used in the results was independently performed by two researchers (AN and UD) through the review of titles and abstracts. 

### 2.5. Methodological Assessment

The methodological quality of the included studies was independently evaluated by two authors (AN and NB) as recommended by the Cochrane Intervention System Evaluation Manual (Cochrane Handbook for Systematic Reviews of Interventions) [11].

If the two researchers disagreed about the methodological assessment, another researcher (SSM) was involved in concluding. The methodological assessment comprised random sequence generation (selective bias), allocation concealment (selective bias), blinding of participants and personnel (performance bias), blinding outcome assessment (detection bias), incomplete outcome data (attrition bias), selective reporting (reporting bias), and other biases.

### 2.6. Strength of Quality across All the Trials

The overall methodological quality of evidence across pooled outcomes was assessed using the Grading of Recommendation, Assessment, Development, and Evaluation (GRADE) guidelines [12]. Study design, bias risk, consistency, directness, precision, and other factors (publication bias, large effect, confounding) were taken into account when determining the evidence for pooled outcomes. The definition of the certainty of the evidence was as follows: (1) high-quality additional research is highly unlikely to alter the confidence in the estimate of effect; (2) moderate-quality additional research is likely to significantly affect the estimate’s confidence and may alter it; and (3) low-quality additional research is highly likely to alter the estimate; or (4) extremely poor quality: the estimate is uncertain.

### 2.7. Quantitative Meta-Analysis

Meta-analysis was performed using RevMan 5.2 [13]. Relative risk (RR) and the 95% confidence interval (CI) were used for binary variables. For continuous variables, mean difference (MD) and 95% CI were used. The results are presented as forest plots, RR or MD, and 95% CI, with *p* < 0.05 being regarded as statistically significant. The χ^2^ and I^2^ tests were performed for clinical heterogeneity in the included studies, and *p* < 0.10 and I^2^ > 50% showed that χ^2^ had statistical differences. A fixed-effects model was utilized if the study results showed low heterogeneity (*p* > 0.10, I^2^ < 50%). The random-effects model was utilized for the meta-analysis in all other cases [11]. To investigate the reasons for high heterogeneity, subgroup analysis and sensitivity analysis were performed, by excluding one study at a time.

### 2.8. Publication Bias

Funnel plots of effect sizes against standard errors for outcomes were examined for asymmetry if there were more than 10 studies that fulfilled the inclusion criteria [14]. The Egger bias test was used as a corresponding statistical test, with *p* < 0.10 indicating asymmetry [15].

### 2.9. Sensitivity Analysis

To verify that the pooled effect sizes were not the result of a single study dominating, data from each study were successively removed, and the remaining data were then reanalyzed to assess the robustness of the pooled estimates for outcomes that included data from three or more studies. 

### 2.10. Trial Sequential Analysis (TSA)

To determine whether our findings were conclusive, TSA was performed on the data using the TSA Module version 0.9.5.10 (Copenhagen trial unit, Denmark, Copenhagen) to calculate the required information size (RIS). The cumulative Z curve was produced using a random-effects model and the DerSimonian–Laird (DL) technique. The goal of TSA was to maintain a 5% overall risk of a type I error [16,17].

## 3. Results

**Figure 1:**
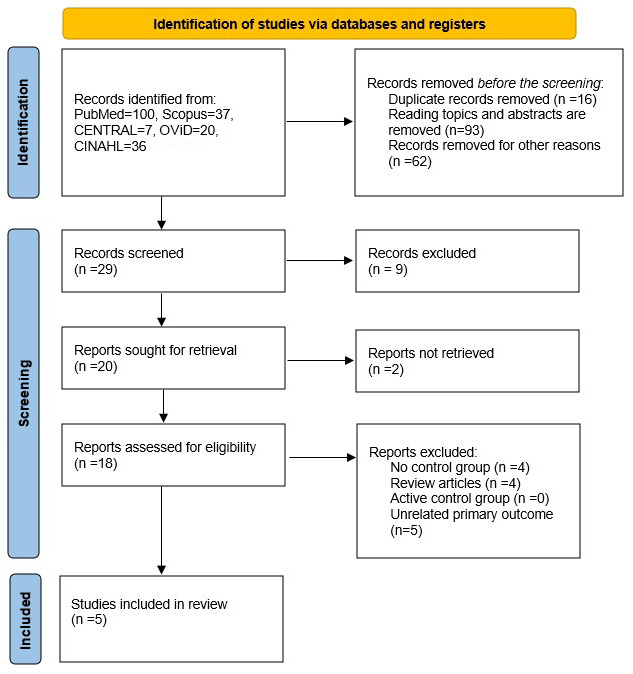
PRISMA flowchart.

A total of 200 articles were identified using the inclusion criteria mentioned above. The details are available in Figure 1. After removing duplicates, and excluding articles that were not relevant, 29 titles were screened, out of which 9 were excluded. From the remaining 20 articles, 2 articles were not retrieved as they were found to not be relevant. Out of the remaining 18 articles, 13 articles were excluded (4 review articles, 4 articles with no control group, and 5 articles with unrelated primary outcome). Finally, 5 articles (RCTs) were selected for a qualitative systematic review and a quantitative meta-analysis [18,19,20,21,22]. Study characteristics and outcome details are summarized in Table 2 and Table 3.

### 3.1. Risk-of-Bias Assessment

**Figure 2:**
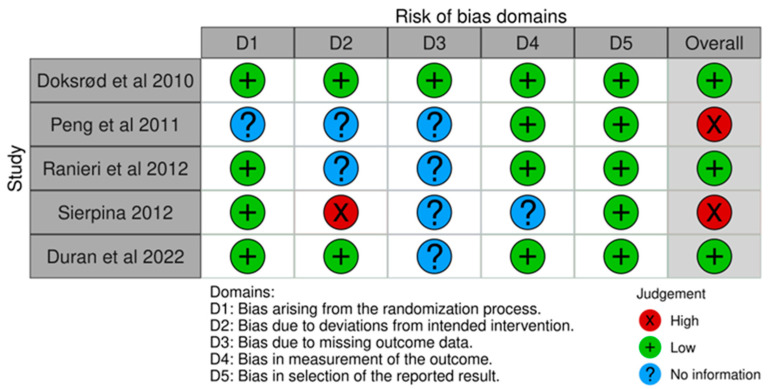
Traffic light plot [18,19,20,21,22].

**Figure 3:**
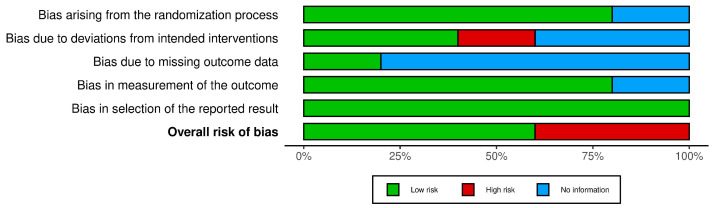
Summary plot.

The risk of bias within the trials according to ROB2 is depicted in Figure 2 (traffic light plot) and Figure 3 (summary plot). The bias from the randomization process was low in four studies [18,20,21,22] and there was no information in one study [19]. Bias due to deviations from intended interventions (allocation concealment) was low in studies [18,22], high in one study [21], and there was no information in two studies [19,20]. Bias arising due to missing outcome data was low in one study [18] and there was no information in four studies [19,20,21,22]. Bias in the measurement of outcome was low in four studies [18,19,20,22] and there was no information in one study [21]. Bias arising due to the selection of reported results was low in all five studies [18,19,20,21,22].

### 3.2. Quality of Evidence

Using the GRADE system, three outcomes were assessed for the quality of evidence (Table 4). The quality of evidence was moderate for laryngospasm, low for airway device failure, and very low for recovery time.

### 3.3. Primary Outcome Meta-Analysis

**Figure 4:**
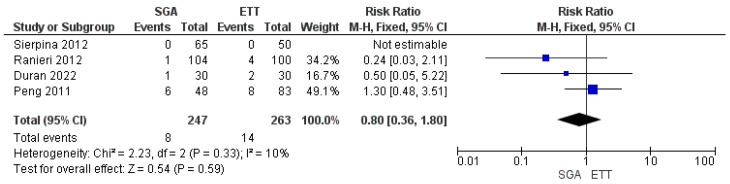
Forest plot showing the comparison of the incidence of laryngospasm between the use of SAD and ETT in children undergoing adenotonsillectomy [19,20,21,22].

Four studies reported laryngospasm as an outcome (247 patients in the SAD group and 263 patients in the ETT group) [19,20,21,22]. A pooled analysis revealed that the incidence of laryngospasm was comparable between the SAD group and the ETT group (RR: 0.80, 95% CI-0.36, 1.80, *p* = 0.59) (Figure 4). A fixed-effect model revealed low heterogeneity (I^2^ = 10%) [GRADE = moderate].

### 3.4. Meta-Analysis of Airway Device Failure

**Figure 5:**
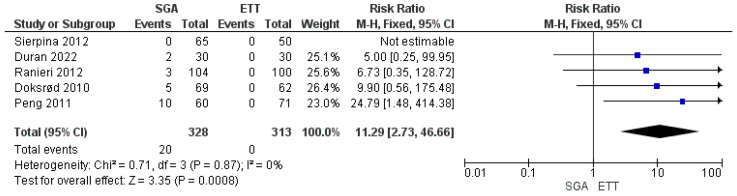
Forest plot showing the comparison of the incidence of airway device failure between the use of SAD and ETT in children undergoing adenotonsillectomy [18,19,20,21,22].

Five studies reported airway device failure, requiring conversion to ETT (328 patients in the SGA group and 313 patients in the ETT group) [18,19,20,21,22]. A pooled analysis revealed that the incidence of airway failure was high in the SAD group (RR: 11.29, 95% CI: 2.73, 46.66, *p* = 0.0008) (Figure 5). A fixed-effect model revealed no heterogeneity (I^2^ = 0%) [GRADE = low].

### 3.5. Meta-Analysis of Postoperative Recovery Time

**Figure 6:**
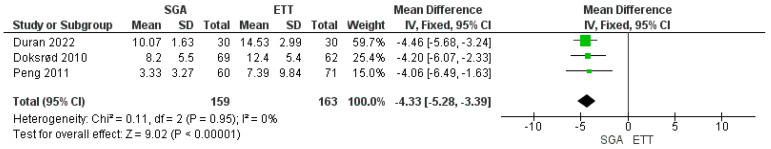
Forest plot showing the comparison of postoperative recovery time between the use of SAD and ETT in children undergoing adenotonsillectomy [18,19,22].

Three studies compared recovery times (159 patients in the SAD group and 163 patients in the ETT group) [18,19,22]. The overall recovery time was significantly less in the SAD group when compared to patients in the ETT group (MD: −4.33, 95% CI: −5.28, −3.39, *p* < 0.0001) (Figure 6). A fixed-effect model revealed no heterogeneity (I^2^ = 0%) [GRADE = very low].

### 3.6. Other Outcomes

Several studies reported other perioperative outcomes. Intubation time and surgical view were reported by Duran et al. [22]. Surgical time, total anesthesia time, and extubation time were reported by Peng et al. [19]. Oxygen saturation was reported by Ranieri et al. [20] and Duran et al. [22]. Hemodynamics (heart rate) and end-tidal carbon dioxide were reported by Doksrød et al. and Duran et al. [18,22]. The duration of surgery was reported by Sierpina et al. and Duran et al. [21,22]. Pooled analysis was not performed for these outcomes as the number of studies reporting them was less.

As the number of included studies was five (less than 10), publication bias was not assessed and therefore funnel plots were not created.

### 3.7. TSA

**Figure 7:**
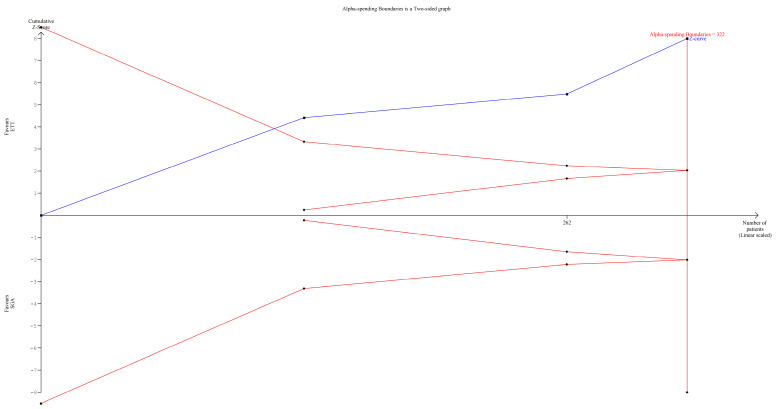
TSA showing the comparison recovery times between the use of SAD and ETT in children undergoing adenotonsillectomy.

TSA was performed only for postoperative recovery time. For the primary outcome, i.e., laryngospasm, the incidence was comparable in both groups. For device failure, the pooled analysis was significant, but as the values were zero in the ETT group, TSA could not be performed. The cumulative z-curve crossed any of the two boundaries, which means that the duration of recovery time was significantly less in the SAD group than in the ETT group (Figure 7).

## 4. Discussion

### Summary of Results

This systematic review and meta-analysis investigated the safety and efficacy of using various SADs in children and adolescents undergoing adenotonsillectomy when compared to ETT. A pooled analysis revealed that the incidence of laryngospasm was comparable with the use of either of the airway adjuncts, with a moderate level of evidence. The failure rate with SADs, i.e., conversion to ETT, was considerably high. The failure of the airway in the ETT group was zero because an ETT is a definitive airway and is unlikely to fail. The overall recovery time with the use of SAD was significantly less. 

Airway management is of paramount importance in pediatric anesthesia [23,24]. This responsibility is even more crucial when the anesthesiologists are sharing the airway with the surgeons, as during adenotonsillectomies. These children have a higher propensity of having respiratory tract infections, mostly viral, and even if they do not have fever, the symptoms like cough and running nose are persistent. Such patients are more prone to perioperative respiratory adverse events (PRAE) like laryngospasm, bronchospasm, and desaturation [25]. 

The use of SADs in various pediatric surgeries became popular as it involves reduced airway stimulation, facilitates faster recovery, and leads to lesser hemodynamic changes, especially during intubation and extubation [26,27]. In a systematic review and meta-analysis investigating the safety and efficacy of LMA in pediatric laparoscopic hernia surgeries, Yang et al. concluded that the use of LMA is safe and leads to lesser anesthesia and recovery time and hence is an appropriate option when compared to ETT [28]. The use of a non-depolarizing muscle for placing LMA is not necessary and depends on the type of surgery and the comfort of the anesthesiologist [29,30]. However, Wu et al. suggested the use of non-depolarizing muscle relaxants can improve surgical conditions and reduce the incidence of adverse events like laryngospasm and bronchospasm [31].

Although many clinicians have started using SAD for various pediatric surgeries, its use in adenotonsillectomies is still considered controversial. Lalwani et al. retrospectively reviewed medical records of 1199 children who underwent adenotonsillectomy from 2003 to 2006, using both LMA and ETT [32]. On analysis, the authors concluded that the use of LMA for pediatric adenoidectomies is linked to a higher rate of complications, primarily from airway obstruction that occurs after LMA insertion or mouth gag placement. They also mentioned that in tonsillectomy, careful patient selection, insertion technique, and avoidance of controlled ventilation may reduce the risk of LMA failure. Surgeons’ ability to operate around the LMA may significantly impact the failure rate.

In a study involving 100 patients from 10 to 35 years undergoing adenotonsillectomy randomized for armored LMA and ETT as the definitive airway for surgery, the authors concluded that an armored laryngeal mask is reliable for performing adenotonsillectomy, provides adequate surgical access for adenotonsillectomy, and is also associated with a lower occurrence of respiratory adverse events like cough, bronchospasm and stridor at recovery. Patients with an armored LMA even demonstrated stable hemodynamics when compared to ETT [33].

In a prospective audit by Thorning et al. comprising 366 day-case pediatric ENT surgeries with LMA, the overall conversion from LMA to ETT was 4.3% (15 patients) [34]. Other than transient desaturation, the rest of the patients were successfully managed over an LMA. In a study comprising 139 pediatric patients (110 LMA, 27 with ETT, and 2 patients with LMA changed to ETT due to ventilatory difficulties) undergoing adenoidectomy, Boroda et al. attempted to investigate the success rate of LMA in these patients as an airway adjunct [35]. The authors concluded that the use of LMA is safe, without any significant adverse events, and also contributes to a reduced operating room time.

Gravningsbråten et al. reported their experience of 1126 pediatric patients (less than 16 years) undergoing office-based adenotonsillectomy using LMA [7]. In this series, one patient was reintubated because of atelectasis, and in six patients the LMA was replaced with ETT due to inadequate ventilation. Two patients underwent reoperation and eight patients were readmitted (two for reoperation and six for observation). They concluded that adenotonsillectomy can be safely performed in an office-based setting using LMA.

In another RCT involving 290 pediatric patients (less than 16 years) undergoing tonsillectomies, Ramgolam et al. compared the occurrence of perioperative respiratory adverse events during the emergence and post-anesthesia care unit phases of anesthesia [36]. On analysis, the authors concluded that there was no evidence for a difference in the timing of the LMA removal on the incidence of respiratory adverse events during the emergence and post-anesthesia care unit phases. However, in the post-anesthesia care unit solely, awake removal was associated with significantly more respiratory adverse events than deep removal. In a retrospective study comprising 179 pediatric patients who underwent adenotonsillectomy with LMA, Eguia et al. concluded that the use of LMA led to an overall reduced intraoperative time [37]. 

However, there was a study by Gehrke et al. in which the authors retrospectively analyzed pediatric adenotonsillectomy in around 1500 patients (683 in the LMA group and 849 in the ETT group) [38]. The authors reported that in at least 10 percent of cases, LMA was replaced with ETT and that the complications were more in the LMA group than ETT group. Based on the results of their study, the authors did not support the use of LMA as an airway device for pediatric adenotonsillectomies. The reason why the use of SGA is not used by many anesthesiologists is due to the fear of adverse events and also that the surgeons might find it difficult to operate with the SGA inside. However, the surgeons can be reassured about this, and in case of difficulty, an ETT can always be placed. The results of the meta-analysis also show that the incidence of respiratory adverse events is similar to SGA when compared to ETT. 

The strength of this systematic review and meta-analysis is that only RCTs were included in the review. The overall heterogeneity of the studies included was low, but the level of evidence was moderate to low. The limitation was a relatively small number of studies could be analyzed. TSA confirmed that the postoperative recovery time was less with the use of SAD than with ETT. The SADs used were of different types in the included studies. Meta-analysis could be performed for three outcomes only as many outcomes were inconsistently reported. 

## 5. Conclusions

The present systematic review and meta-analysis suggest that when SAD is used for adenotonsillectomy, there is lesser postoperative time and comparable incidence of laryngospasm, but a higher chance of change over to ETT. It is recommended to use SAD for these surgeries by careful selection of patients and also based on the experience of the anesthesiologists and surgeons involved. We suggest the conduction of well-designed and adequate power studies in the future, with a robust methodology to establish the ideal SAD that could be used with a lesser incidence of failure.

## Figures and Tables

**Table 1:** Search details for various databases.

PubMed search details: (100)	((((“supraglottal” [All Fields] OR “supraglottic” [All Fields]) AND (“airway” [All Fields] OR “airway s” [All Fields] OR “airways” [All Fields]) AND (“laryngeal masks” [MeSH Terms] OR (“laryngeal” [All Fields] AND “masks” [All Fields]) OR “laryngeal masks” [All Fields] OR (“laryngeal” [All Fields] AND “mask” [All Fields] AND “airway” [All Fields]) OR “laryngeal mask airway” [All Fields])) OR (“adenoidectomy” [MeSH Terms] OR “adenoidectomy” [All Fields] OR “adenoidectomies” [All Fields]) OR (“tonsillectomy” [MeSH Terms] OR “tonsillectomy” [All Fields] OR “tonsillectomies” [All Fields])) AND (“adenotonsillectomies” [All Fields] OR “adenotonsillectomy” [All Fields]) AND (“anaesthesia” [All Fields] OR “anesthesia” [MeSH Terms] OR “anesthesia” [All Fields] OR “anaesthesias” [All Fields] OR “anesthesias” [All Fields])) AND ((clinicaltrial[Filter] OR randomizedcontrolledtrial[Filter]) AND (2000/1/1:2024/1/10 [pdat]) AND (allinfant[Filter] OR infant[Filter] OR child[Filter] OR adolescent[Filter] OR preschoolchild[Filter]))
Scopus search details: (37)	TITLE-ABS-KEY “Supraglottic Airway” OR “Laryngeal mask airway” AND “Adenoidectomy” AND “Tonsillectomy” OR “Adenotonsillectomy” AND “Anesthesia”
Cochrane library: (7)	“laryngeal mask airway” in Title Abstract Keyword AND “adenoidectomies” in Title Abstract Keyword AND “tonsillectomies” in Title Abstract Keyword AND “anesthesia” in Title Abstract Keyword AND “pediatric” in Title Abstract Keyword (Word variations have been searched) “laryngeal mask airway”:ti,ab,kw AND “adenoidectomies”:ti,ab,kw AND “tonsillectomies”:ti,ab,kw AND “anesthesia”:ti,ab,kw AND “pediatric”:ti,ab,kw (Word variations have been searched)
CINAHL: (36)	Search terms: supraglottic airway OR laryngeal mask airway AND (adenoidectomy or tonsillectomy or adenotonsillectomy) Limiters—Publication Date: 20000101–20241231 Expanders—Apply equivalent subjects Narrow by SubjectAge:—adolescent: 13–18 years Narrow by SubjectAge:—infant: 1–23 months Narrow by SubjectAge:—child: 6–12 years Narrow by SubjectAge:—child, preschool: 2–5 years Narrow by SubjectAge:—all child Search modes—Boolean/Phrase
OViD: (20)	Supraglottic airway* laryngeal mask airway* adenoidectomy*tonsillectomy*pediatric*adolescent.mp. [mp = title, abstract, full text, caption text]

**Table 2:** Study characteristics.

Author/Year	Study Design	Study Population	Group I (SGA)	Group II (ETT)	Sample Size	Age Group (Years)	Type of SGA Used	Outcomes	Findings
Doksrød et al./2010 [18]	RCT	Pediatric and adolescent	69	62	131	3–16	R-LMA	To compare pain, nausea, and respiratory irritation with R-LMA and ETT. Time spent in the operating room after surgery.	Significantly lower pain scores with R-LMA at 4 h (*p* = 0.015) only and not significant at other times till 24 h Lesser time spent after surgery in the operating room (4.2 min lesser) with R-LMA (*p* = 0.001) Nausea was comparable in both groups. In patients in ETT group and five patients in whom R-LMA was changed to ETT, airway irritation was higher (*p* < 0.02).
Peng et al./2011 [19]	RCT	Pediatric	60	71	131	2–12	F-LMA	Primary outcome: to compare the incidence of laryngospasm. Secondary outcomes: anesthesia, operative and recovery time.	Incidence of postoperative laryngospasm between LMA and ETT (12.5% and 9.6%) was similar (*p* = 0.77). Time from surgery end to extubation was significant with LMA (*p* = 0.01) by 4.06 min. Mean surgical time and postoperative recovery times were comparable (*p* = 0.15 and *p* = 0.49).
Ranieri et al./2012 [20]	RCT	Pediatric	104	100	204	2–10	LMA Unique	To evaluate the levels of blood oxygenation and the occurrence of respiratory complications during adenotonsillectomies.	The use of LMA resulted in a lower intraoperative SpO2, compared to using an ETT (at induction: *p* > 0.25, after establishing operative field: *p* < 0.001, at end of surgical procedure: *p* = 0.037, 3 min after extubation: *p* < 0.001 On admission to recovery room: *p* < 0.001). Respiratory complications (bronchospasm, laryngospasm, stridor, breathing noise, regurgitation) were comparable.
Sierpina et al./2012 [21]	RCT	Pediatric and adolescent	65	50	115	2–18	F-LMA	Safety, duration of surgery, and patient comfort	Less coughing and gagging with LMA (*p* = 0.002). Safety and various postoperative outcomes were comparable.
Duran et al./2022 [22]	Comparative study	Pediatric	30	30	60	2–12	F-LMA	Intubation and recovery time, view of the surgical field, SpO2, ETCO2, heart rate, and airway pressure.	Intubation and recovery time were shorter in the F-LMA group than in the ETT group, in minutes (16.93 ± 4.84 s vs. 23.93 ± 8.74 s; and 10 ± 2 vs. 14.5 ± 3; *p* < 0.001). The airway pressures were significantly lower in the F-LMA group than in ETT (*p* < 0.001).

RCT: randomized controlled trials, LMA: laryngeal mask airway, ETT: endotracheal tube.

**Table 3:** Outcome data in all the included studies.

S. No.	Study	Airway Device Used	Total Patients	Laryngospasm	SGA Failure	Recovery Time (Minutes)
1	Doksrød et al./2010 [18]	SAD	69	--	5	8.2 ± 5.5
		ETT	62	--	0	12.4 ± 5.4
2	Peng et al./2012 [19]	SAD	48	6	10	3.33 ± 3.27
		ETT	83	8	0	7.39 ± 9.84
3	Ranieri et al./2012 [20]	SAD	104	1	3	--
		ETT	100	4	0	--
4	Sierpina et al./2012 [21]	SAD	65	0	0	--
		ETT	50	0	0	--
5	Duran et al./2022 [22]	SAD	30	1	2	10.07 ± 1.63
		ETT	30	2	0	14.53 ± 2.99

SAD: supraglottic airway device; ETT: endotracheal tube; --: This outcome was not evaluated in those studies.

**Table 4:** GRADE strength of evidence.

Certainty Assessment							№ of Patients		Effect		Certainty	Importance
№ of Studies	Study Design	Risk of Bias	Inconsistency	Indirectness	Imprecision	Other Considerations	Supraglottic Airway	Endotracheal Tube	Relative (95% CI)	Absolute (95% CI)		
**Laryngospasm**												
4	randomized trials	Serious ^a^	Serious ^b^	not serious	not serious	all plausible residual confounding would reduce the demonstrated effect	8/259 (3.1%)	14/251 (5.6%)	**RR 0.00** (0.32 to 1.92)	-- per 100 (from 4 fewer to 5 more)	⨁⨁⨁◯ Moderate	IMPORTANT
**Airway device failure**												
5	randomized trials	Serious ^a^	Serious ^b^	not serious	Serious ^b^	publication bias strongly suspected all plausible residual confounding would reduce the demonstrated effect ^c^	10/328 (3.0%)	0/313 (0.0%)	**RR 0.00** (2.73 to 46.66)	-- per 100 (from 0 fewer to 0 fewer)	⨁◯◯◯ Very low	IMPORTANT
**Time spent in OR**												
3	randomized trials	Serious ^a^	Serious ^d^	Serious ^d^	Serious ^d^	strong association all plausible residual confounding would reduce the demonstrated effect	159	163	-	**0** (0 to 0)	⨁⨁◯◯ Low	IMPORTANT

CI: confidence interval; RR: risk ratio. a. There was either no information about various types of bias or the bias was high in the included studies. b. The results were inconsistent between the included studies. c. There was a disparity between number of studies with positive and negative outcome. d. The definition of the outcome was not uniform.

## Data Availability

The data used to support the findings of this study are available upon request to the corresponding author.
